# Assessing Habitat Use by Snapper (*Chrysophrys auratus*) from Baited Underwater Video Data in a Coastal Marine Park

**DOI:** 10.1371/journal.pone.0136799

**Published:** 2015-08-28

**Authors:** Maria A. Terres, Emma Lawrence, Geoffrey R. Hosack, Michael D. E. Haywood, Russell C. Babcock

**Affiliations:** 1 Department of Statistics, North Carolina State University, Raleigh, North Carolina, United States of America; 2 Digital Productivity Flagship, CSIRO, Dutton Park, Queensland, Australia; 3 Digital Productivity Flagship, CSIRO, Castray Esplanade, Hobart, Tasmania, Australia; 4 Oceans and Atmosphere Flagship, CSIRO, Dutton Park, Queensland, Australia; The Australian National University, AUSTRALIA

## Abstract

Baited Underwater Video (BUV) systems have become increasingly popular for assessing marine biodiversity. These systems provide video footage from which biologists can identify the individual fish species present. Here we explore the relevance of spatial dependence and marine park boundaries while estimating the distribution and habitat associations of the commercially and recreationally important snapper species *Chrysophrys auratus* in Moreton Bay Marine Park during a period when new Marine National Parks zoned as no-take or “green” areas (i.e., areas with no legal fishing) were introduced. BUV studies typically enforce a minimum distance among BUV sites, and then assume that observations from different sites are independent conditional on the measured covariates. In this study, we additionally incorporated the spatial dependence among BUV sites into the modelling framework. This modelling approach allowed us to test whether or not the incorporation of highly correlated environmental covariates or the geographic placement of BUV sites produced spatial dependence, which if unaccounted for could lead to model bias. We fitted Bayesian logistic models with and without spatial random effects to determine if the Marine National Park boundaries and available environmental covariates had an effect on snapper presence and habitat preference. Adding the spatial dependence component had little effect on the resulting model parameter estimates that emphasized positive association for particular coastal habitat types by snapper. Strong positive relationships between the presence of snapper and rock habitat, particularly rocky substrate composed of indurated freshwater sediments known as coffee rock, and kelp habitat reinforce the consideration of habitat availability in marine reserve design and the design of any associated monitoring programs.

## Introduction

No-take marine reserves are spatial closures where all forms of extraction are banned. They have been promoted as assisting in reducing over-fishing [1], conserving marine biodiversity, restoring populations of endangered species [2] and in restoring lost trophic structure [3]. Over the past few decades, marine reserves have been increasingly recognized as a key component of marine conservation strategies [4, 5]. While marine reserves are generally found to be an effective means of achieving positive conservation outcomes, their success in this regard depends on a range of interacting factors such as being no take, well enforced, old (at least 10 years), large (greater than 100km^2^), and isolated by deep water or sand [6], as well as containing suitable habitat types [7]. As with any re-allocation of resources, the implementation of marine reserves is socially and economically complex with potential tradeoffs. Given the uncertainty of the ecological effects of marine reserves and their often contentious nature [8] the evaluation of the effectiveness of marine reserve conservation outcomes forms an important part of most marine conservation strategies, and ideally incorporates adaptive management [9]. The increasing number and extent of marine reserves reinforces the need for efficiently designed monitoring and information systems that inform their management.

Marine reserve protection may particularly benefit actively fished species that exhibit high site fidelity, such as snapper (*Chrysophrys auratus*) in southeast Queensland, Australia [10, 11], which are the most abundantly harvested rock reef species in this region [12]. Snapper are an iconic species actively targeted by both professional and recreational fishers, and in the past the sustainability of stocks have been of concern [13, 14]. Snapper are a suitable candidate for marine reserve protection because of relatively localised movement patterns in their behavior [15]. No-take areas have been successful in increasing both biomass and abundance of snapper, most notably in New Zealand at the Poor Knights [16], Leigh [17], Hahei and Tawharanui Marine Reserves [18]. The effectiveness of no-take areas in protecting snapper populations has been more variable in Australia however [19, 20].

Ideally no-take areas should protect the preferred habitat of a conserved species. Snapper populations likely contain heterogeneous behavioral patterns with habitat preferences that change with season and age [21]. Adult snapper have been suggested to be residential over rocky reef habitat relative to soft sediment habitats that have fewer resources [22, 23], which suggests that the availability of suitable habitat should be accounted for when determining the effect of protection zones for snapper. Complex benthic habitats have been shown to influence juvenile snapper distribution and habitat use in New Zealand [24], but juvenile snapper population densities in Moreton Bay (located in southeast Queensland, Australia) have been suggested to not significantly differ across habitat types (sand, mud, rubble and soft coral/algal beds) [25].

Programs designed to evaluate the effectiveness of marine reserves, as with any evaluation and monitoring programs, need to be robustly effective and have an efficient means of determining ecological and management outcomes. Because the evaluation of marine reserve effects requires spatial comparisons, it is important to include the consideration of other sources of variation in the spatial distribution of species of interest. For example, fish are often highly social, forming schools, and often prefer specific habitats. Such factors complicate the assessment of the effectiveness of a marine park for protecting a species of interest. Due consideration of these effects in the statistical data evaluation have the potential to increase the effectiveness of evaluation and monitoring programs. Here we demonstrate the utility of statistical analyses that incorporate both spatial and habitat level effects on fish presence of the commercially and recreationally targeted fish *Chrysophrys auratus* in the Moreton Bay Marine Park, established in 1997 and re-zoned extensively in 2009.

To gain an understanding of the effects and potential benefits of the no-take marine reserve zones, a monitoring program was designed to sample the original (1997) reserves and a sample of the newly designated protected reserves (now Marine National Park Zones [MNP]) as well as adjacent areas open to fishing but similar in depth and habitat. One aspect of the program was the use of stereo Baited Underwater Video systems (BUVs) [10, 26] for monitoring species occurence and size. BUVs have become a common method of data collection for this purpose due to their non-destructive nature, repeatability and far greater depth range of deployment compared to surveys by scuba divers [27]. Recent uses of BUVs for monitoring in marine parks include monitoring for individually targeted species and reef fish assemblages at different spatial and temporal scales [19, 21, 28].

Previous model-based analyses of BUV data have used generalized linear models [29], mixed effects models [30, 31] and more recently Bayesian zero-inflated GLMMs [32]. The most common choice of response variable is the so-called MaxN count [21, 31, 33, 34], which is the largest number of individuals of a given species observed in any single video frame. These, however, did not explicitly test for and/or directly incorporate spatial autocorrelation in species distribution models [35], although some researchers instead advocate a minimum distance between BUV sites [7, 36] to minimize the possibility of double counting fish [37]. These minimum distances can lead to low levels of replication in small reserves reducing effectiveness (ability to detect effects). In addition, this approach does not account for spatial dependence that arises because of unmeasured covariates that may be shared across different sites. For example, fine-scale habitat features may be similar for proximate locations, but could be difficult to observe, quantify or model directly. The assumption that each site is an independent observation is often unverified and could bias the results of analyses [38].

With these considerations in mind, we implement a spatial logistic model for snapper presence. We fit the model in a fully Bayesian framework so as to comprehensively quantify the associated uncertainty [39]. Presence-absence is used as an indicator of habitat suitability and species occurrence, and we explicitly incorporate spatial dependence and available habitat covariates into our modeling framework using a spatial process model to assess how spatial dependence affects relationships between snapper presence, marine park boundaries, environmental covariates such as depth, and key habitat covariates. The habitat covariates include kelp and rock, where we also focus particularly on so-called coffee rock. This is a form of sedimentary rock composed of compressed freshwater sediments that occurs along the coast of southeast Queensland [40], and given that rocky reef habitats are important for snapper [21, 23] it may be an important habitat feature to consider in the design of marine parks within this region.

## Materials and Methods

### Study Area

The original Moreton Bay Marine Park (MBMP) was established in 1997 to provide permanent preservation of biological biodiversity and natural condition in no-take protection zones to the maximum extent possible [41]. The park was re-zoned in 2009 on the basis of an extensive review. The result was 34 Marine National Park (MNP) Zones designated as no take areas, which we refer to as “green” zones, ranging in size from 38 to 12541 hectares designed to ensure adequate protection of each of the 16 habitat types identified within the Marine Park [42]. The Park boundaries encompass the whole of Moreton Bay and extend several kilometres offshore from the Barrier Islands (Moreton, and north and south Stradbroke Islands) that enclose the eastern side of Moreton Bay. Seven of the MNP Zones lie to outside of Moreton Bay, to the east of the barrier islands. Two of these offshore MNPs were the subject of this study: MNP04 and MNP10, encompassing 0.78 km^2^ and 87.15 km^2^ respectively. While these MNPs are relatively small, they were deemed adequate for the primary goal of protecting the unique habitats found in the region, with the protection of reef-associated species considered to be a secondary benefit.

### Baited Underwater Video

As this study involved surveying fish populations inside marine reserves, the use of conventional extractive sampling techniques such as netting or line fishing were not an option. Underwater Visual Census (UVC) has been widely used in similar studies however repetitive scientific diving is generally limited to 20 m and the depth of some of our sites (up to 47 m) precluded using divers. Instead, we used stereo Baited Underwater Video (BUV) units supplied by SeaGIS Pty. Ltd (www.seagis.com.au) to enable length measurements of individual fish. These systems consisted of a pair of digital video cameras (Panasonic NVGS330) with wide-angle lenses in waterproof housings mounted on a galvanised steel frame. The frames were fitted with breakaway legs in the event of fouling on the seabed and were able to be loaded with a variable amount of ballast if required under high current conditions. A rotating flashing diode mounted on a pole such that it was visible from both cameras facilitated synchronisation of the two stereo images during video analysis. A bait bag (220 × 150 mm; 10 mm mesh size) made from plastic gutter guard was mounted on a PVC conduit pole and fixed approximately 1.2 m in front of the cameras.

Prior to deployment each video camera was loaded with a new mini DV tape and the bait bag was filled with pilchards (approximately 750 g). The pilchards were crushed in order to maximise dispersal of the fish oil and flesh. Each BUV had a surface buoy attached with 8 mm rope to facilitate recovery. BUVs were deployed for 45 min as previous research has demonstrated that a deployment time of at least 30 minutes is necessary to record the majority of the fish species [43, 44].

### Data and Collection

The part of the MBMP monitoring program that this study focused on was designed to sample two original reserves (MNP04 and MNP10), two new reserves (extensions to MNP04 and MNP10) and selected areas open to fishing identified as having similar habit, bathymetry and oceanographic conditions to the selected reserves. While each of the MNPs are contiguous in shape there were generally multiple smaller controls for each reserve, resulting in a clustering of sites.

MNP04 surrounds Flinders Reef which is a shallow reef, breaking the surface in points, and is characterised by extensive coral reef communities in shallow water with at least 115 scleractinian species recorded and over 20% cover of coral [45]. Reefs in MNP10 reach their shallowest extent at around 12-14m depth with most of the reef habitat distributed at depths between 20-30m. On these reefs the temperate kelp *Ecklonia radiata* is the dominant habitat forming alga [42], likely encouraged by cooler upwelled waters that are experienced at depths of 20m or more at these sites (R. Babcock, personal observations).

To select the sites a 50m grid was placed over the spatial extent of the reserves and controls. The sites were selected using Generalised Random Tessellation Stratified sampling [46], ensuring a maximum of one site per 50m grid. The GRTS method is a design-based method for selecting sites in a manner that achieves spatial balance with randomness but less clustering than conventional random sampling. Although we did not intend to use design-based analysis on the data collected, this method was implemented as an objective way of selecting sites with good spatial balance.

Data were collected at 5 time points (3 winters and 2 summers) between 2008 and 2010, during this period (1 March 2009) some areas were newly classified as green (no-fishing) zones. At each site a BUV is deployed for 45 minute sessions in one or more seasons and years. A total of 174 sites were monitored, with most sites visited at multiple time points, resulting in a total of 536 observations. Covariates at each site include depth, reef/non-reef, conservation status (green:old, green:new or open), winter/summer, and visibility range. Visibility, season, and status vary in time at some or all replicated sites and are incorporated into time-varying covariates for these sites. For example, the status covariate at a site with deployments both before and after the closure took effect would be classified as open for the earlier deployments and would be classified as green:new for the later deployments.

**Fig 1:**
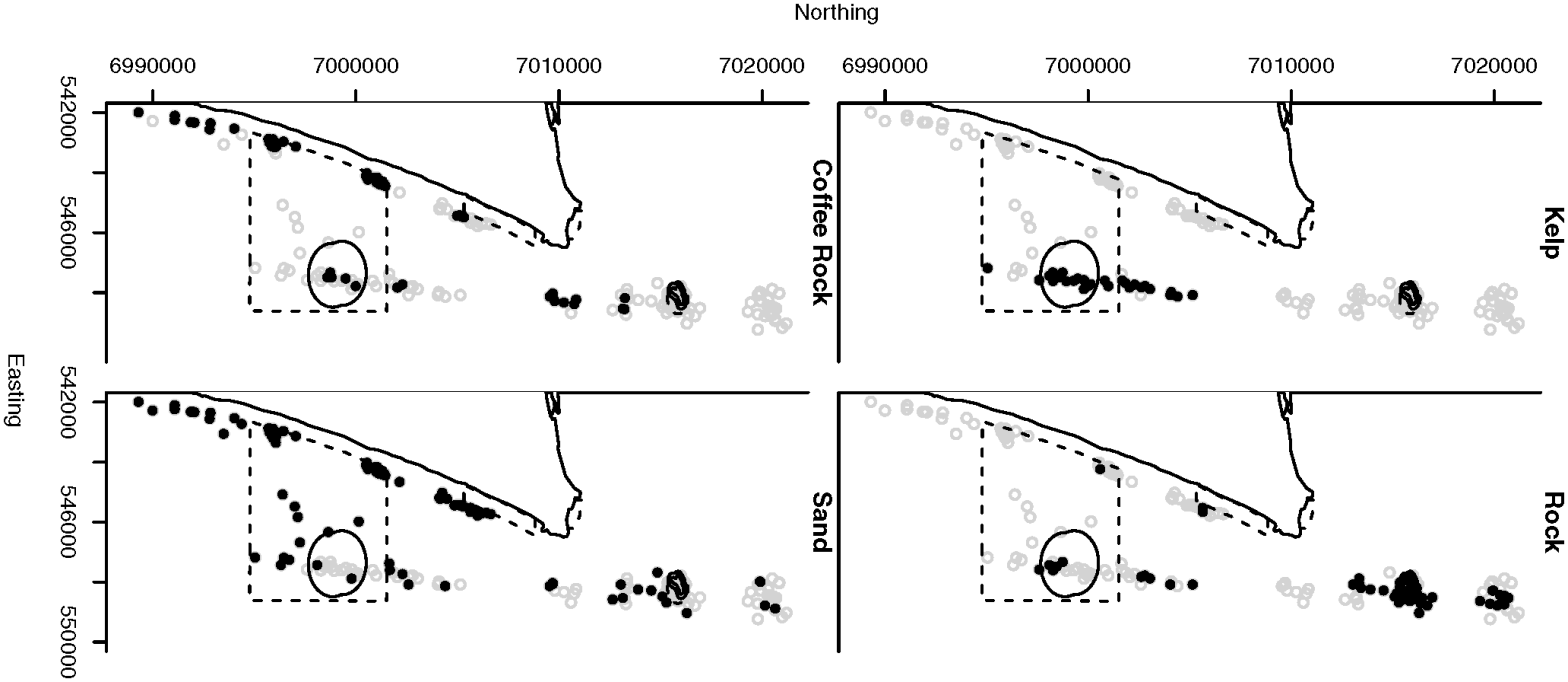
BUV site locations where habitat types were present. The open grey circles indicate sites where that habitat variable did not cover at least 5% of the site and solid black circles indicate sites where that habitat covered at least 5% of the site. The solid line indicates the Green:old areas while the broken line indicates Green:new areas. Sites outside the bounded areas are open to fishing. MNP04 is the green area in the North and MNP10 in the South. The easting and northing axes are measured in units of meters.

We recorded video footage of the seabed around each BUV site using a Sea-Drop (http://www.Seaviewer.com) underwater video camera to derive estimates of percent cover of a variety of substrate, algal and epibenthic categories. The camera was lowered to approximately 1 m above the seabed 50 m away from the site and then towed at 1.5 knots towards the site and for 50 m on the opposite side of the site. The video was recorded directly onto mini DV tapes and later digitised in the laboratory. The video was analysed using TransectMeasure (SeaGIS Pty. Ltd.) software by stepping through and pausing the video footage at 5 s intervals. At each interval a grid of 6 dots was overlaid on the video image and the operator would identify the substrate and any epibenthos and/or algae underlying the dot. Fig 1 shows the BUV sites where some of the key habitats were observed.

Within a given sampling season adjacent replicate sites were usually separated by a minimum of 250 m to avoid the possibility of the same fish visiting adjacent BUVs during the sampling period. In some cases, due to a paucity of the appropriate habitat, it was necessary to locate sites ∼ 100 m apart; in these cases the deployments were not done on the same day. While many sites were replicated for different seasons and years, there were also several sites that were not replicated. In our modeling approach it is necessary to consider all locations where BUVs were deployed regardless of season. Across all sites the minimum inter-site distance was 20 m (corresponding to two sites measured in different seasons), the mean distance was 10,820 m, and the maximum distance was 32,660 m. When all these sites are mapped there are several clusters of sites, with sites tightly located within each cluster due to the proximity of sites measured in different seasons. Note, multiple BUVs were never deployed simultaneously. The geographic locations are considered under the UTM Zone 56 South coordinate system, such that the coordinates are measured in meters.

A Marine Park Permit to carry out all field work was obtained from the Environment Protection Agency, Queensland Parks & Wildlife Division, Moreton Bay Region.

Table 1 provides observed presence percentages for the different status and season. The proportion of BUV deployments in which snapper were observed was slightly higher in green zones and during winter.

**Table 1:** The number of observations *N* with each of the attributes and the percentage of these observations where snapper presence was observed.

	Green:Old	Green:New	Open	Winter	Summer
*N*	108	94	349	301	250
% Present	57.4	53.2	47.0	55.8	43.2

Note, the processed data used for analysis are provided as supporting information, and the raw data can be made available upon request.

### Statistical Model

We fitted two Bayesian logistic regressions to model presence-absence of the snapper species. Both regressions incorporate time-varying green zone variables indicating whether the location was a designated green zone at the time of the observation. This variable is recorded as “green: old” indicating the location was included in the original 1997 reserve designation, “green:new” indicating the location was added to the reserve designation in 2009, or “open” indicating that the location was not in a reserve area. Our interest is in understanding whether these green zone reserve areas had an effect on snapper presence. The regressions additionally control for depth (m), season (“winter” or “summer”), visibility (m) which influences the volume of water censused for fish, and the presence or absence of rock, kelp, sand or coffee rock at the site. It is expected that the composition of the sea floor will impact the desirability of the location as a habitat for the snapper.

In addition, it was of interest to assess any spatial dependence present in these data and to understand what impact it may have on our resulting inference regarding the impact of green zones. To accomplish this we incorporated spatial random effects into one of the regressions, referred to as the “Spatial Model” and compared the results to those from the “Non-Spatial Model” where these effects were not incorporated.

Specifically, we used the following model framework,

$$\begin{array}{ccc} P(Y_{t}\left(s\right)=1) & = & p_{t}\left(s\right)\\ \text{logit}(p_{t}\left(s\right)) & = & X_{t}\left(s\right)\beta +\eta \left(s\right) \end{array}$$ (1)

where *p*_*t*_(*s*) is the probability of presence in year *t* at location *s*, *X*_*t*_(*s*) is the covariate vector in year *t* at location *s*, and *η*(*s*) is a spatial random effect at location *s*. In the case of the Non-Spatial Model the spatial random effect *η*(*s*) was excluded from Eq (1).

The static coefficients in the model, *β*, suggest that the presence or absence of snapper relates to the covariates in the same way across time and space. However, the spatial random effects *η* allow for location specific adjustments, capturing spatial dependence not otherwise explained by the covariates. The spatial effects were modeled as a realization from a Gaussian process. That is, ***η*** = (*η*(*s*_1_), …, *η*(*s*_*N*_))′ were assumed to follow a Multivariate Normal distribution with a covariance structure that encourages stronger correlations for locations close to one another. Note, the spatial effects were introduced in the transformed mean thereby encouraging the probabilities of presence to be similar at proximate locations [47].

For data with a longer time series, additional structure could be incorporated into the model to capture temporal trends. In our case temporal variation across 2.5 years is expected to be minimal and was assumed to be sufficiently explained by the temporal covariates, *X*_*t*_(*s*).

To complete the model specification we assumed prior distributions for each of the model parameters:

$$\begin{array}{ccc} \beta_{i} & ∼ & N(0,2.5),\;i=0,\ldots ,9\\ 1/\phi & ∼ & Unif(50,1500)\\ \sigma^{2} & ∼ & InvGam(2,2)\\ \eta |\phi ,\sigma^{2} & ∼ & N(0,\sigma^{2}\Sigma \left(\phi \right)) \end{array}$$

where the prior mean and variance were the same for all *β*_*i*_ and *σ* ^2^Σ_*ij*_(*ϕ*) = *Cov*(*η*(*s*_*i*_), *η*(*s*_*j*_)) = *σ* ^2^ exp(−*ϕ*∣∣*s*_*i*_ − *s*_*j*_∣∣). The prior on the coefficients *β*_*i*_ follows suggestions in the literature [48], effectively placing a prior on the resulting probabilities *p*_*t*_(*s*) such that they are distributed fairly uniformly between 0 and 1. In the spatial modeling community it is well known that the ratio of *σ* ^2^ and *ϕ* is well identified but the parameters separately are not [47]. This is particularly true for binary data where little information is available on the spatial dependence. To produce well behaved parameters a typical approach is to place a broad prior on *σ* ^2^ and a more informative prior on *ϕ*. The effective range is defined as the distance where the spatial correlation is 0.05 [47], which is approximately 3/*ϕ* for this model. The prior on *ϕ* is selected to place most of the prior mass in a region producing sensible values for the effective range.

The models were fitted in a Bayesian framework using Markov chain Monte Carlo in JAGS and R (code is provided as supporting information). Analyses were based on 5000 posterior samples, after a burn in of 1000 iterates. A thinning of every second iterate was used in the non-spatial model, and a thinning of every tenth iterate was used in the spatial model. The depth and visibility covariates were standardized prior to model fitting, allowing for comparison of covariate effects and improving mixing of the MCMC chains.

### k-fold Cross Validation

To enable further confidence in our results we checked their sensitivity using a 5-fold cross validation. Each location was randomly assigned to one of five data subsets or folds, placing all observations at a location in the same subset. The analysis was then repeated five times, each time leaving out one of the five subsets for out-of-sample prediction and fitting the models to the four remaining subsets. By assigning folds based on location we were able to test the predictive capability of the Spatial Model in situations where the spatial random effect must be sampled for each new location.

### Measures of Comparison

We compared the Spatial and Non-Spatial Models using three criterion: the posterior mean log likelihood (LL), the mean squared error (MSE) and the area under the curve (AUC). The AUC statistic relates to receiver operating characteristic (ROC) plots commonly used to assess the sensitivity and specificity of a proposed model [49]. There is an ROC curve corresponding to each model, where each point on the curve plots the false positive rate against the true positive rate. If the model is classifying the sites as presence/absence successfully, then the false positive rate should be low and the true positive rate should be high. As such, a curve that reaches to the upper left corner of the figure indicates a strong model. AUC is simply the area under the curve in the ROC plot, and a value close to 1 indicates a good fit. The LL, MSE and ROC statistics were first computed for within-sample predictions for the full data set. Then, for the k-fold cross validation, they are computed for out-of-sample prediction on the held out fold under each of the five analyses.

## Results

### Analysis on Full Data

**Fig 2:**
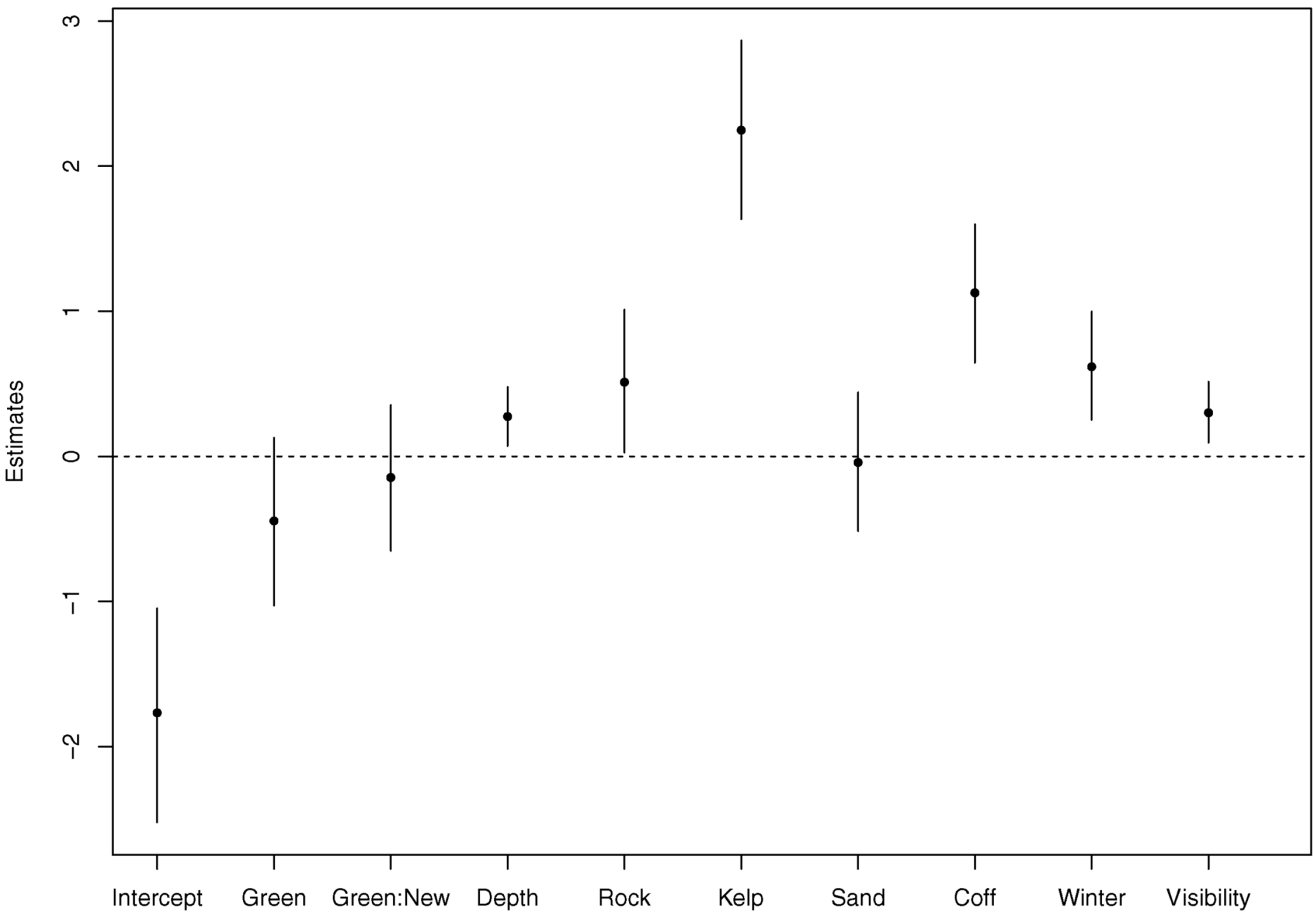
Estimates for the coefficients in the Non-Spatial model. Posterior means and 95% credible intervals.

The fitted parameters and corresponding 95% credible intervals are provided for the Non-Spatial Model in Fig 2 and for the Spatial Model in S1 Fig. (Detailed results are provided in S1 Table). The resulting parameter estimates are generally similar, identifying five of the same covariates as significant at the 0.05-level (95% credible intervals not overlapping zero). These covariates are presence of rock, presence of kelp, presence of coffee rock, season, and visibility. The magnitudes of these coefficients are slightly larger under the Spatial Model, although the general interpretations remain the same. Finally, depth is the only variable identified as significant under the Non-Spatial Model and not identified under the Spatial Model, although it maintains the same sign. In some cases the uncertainties associated with coefficients are larger in the Spatial Model, which is not surprising since we have introduced an unknown spatial random effect for each location that is not present in the Non-Spatial Model. These unknown random variables introduce an additional source of uncertainty.

Exponentiating the coefficients in a logistic regression allows for inference regarding the implied effects on the odds of presence. In this way, we interpret the effect of the covariates under the Non-Spatial Model as follows. The odds of presence in old green zones are 0.67 (95% credible interval: 0.36,1.14) times that in open zones and in new green zones 0.89 (0.52, 1.43) times that in open zones, though neither result is significant (credible intervals overlap 1). In addition, the odds of presence are 1.32 (1.07, 1.61) times higher for every additional standardized unit of depth, 1.72 (1.03, 2.75) times higher when rock is present, 9.96 (5.13, 17.58) times higher in the presence of kelp, 0.99 (0.60, 1.55) times higher in the presence of sand i.e. unaffected, 3.18 (1.91, 4.95) times higher in the presence of coffee rock, 1.89 (1.29, 2.71) times higher in winter than in summer, and 1.36 (1.10, 1.67) times higher for every additional standardized unit of visibility.

Similarly, we interpret the effect of the covariates under the Spatial Model as follows. The odds of presence in the old green zones are 0.81 (95% credible interval: 0.26, 2.06) times the open zones and in the new green zones, 0.95 (0.45, 1.74) times the open zones. Neither of these parameters are significant in the model. The odds of presence are also 1.22 (0.91, 1.61) times higher for every additional standardized unit of depth (not-significant), 2.74 (1.25, 5.52) times higher when rock is present, 15.69 (4.56, 42.51) times higher when kelp is present, 0.81 (0.37, 1.50) times higher when sand is present (not-significant), 3.83 (1.76, 7.44) times higher when coffee rock is present, 2.10 (1.37, 3.10) times higher in winter than in summer, and 1.37 (1.07, 1.74) times higher for every additional standardized unit of visibility.

In the Spatial Model, the parameter *ϕ* implies a median effective range of 1979.51 (mean of 2141.57) meters with a 95% highest posterior density (HPD) credible interval of (538.02, 4171.83) (S1 Table, S2 Fig). The prior and posterior for the effective range, 3/*ϕ*, are provided in S2 Fig, with the skewed shape suggesting the use of the median and HPD interval as summaries.

**Fig 3:**
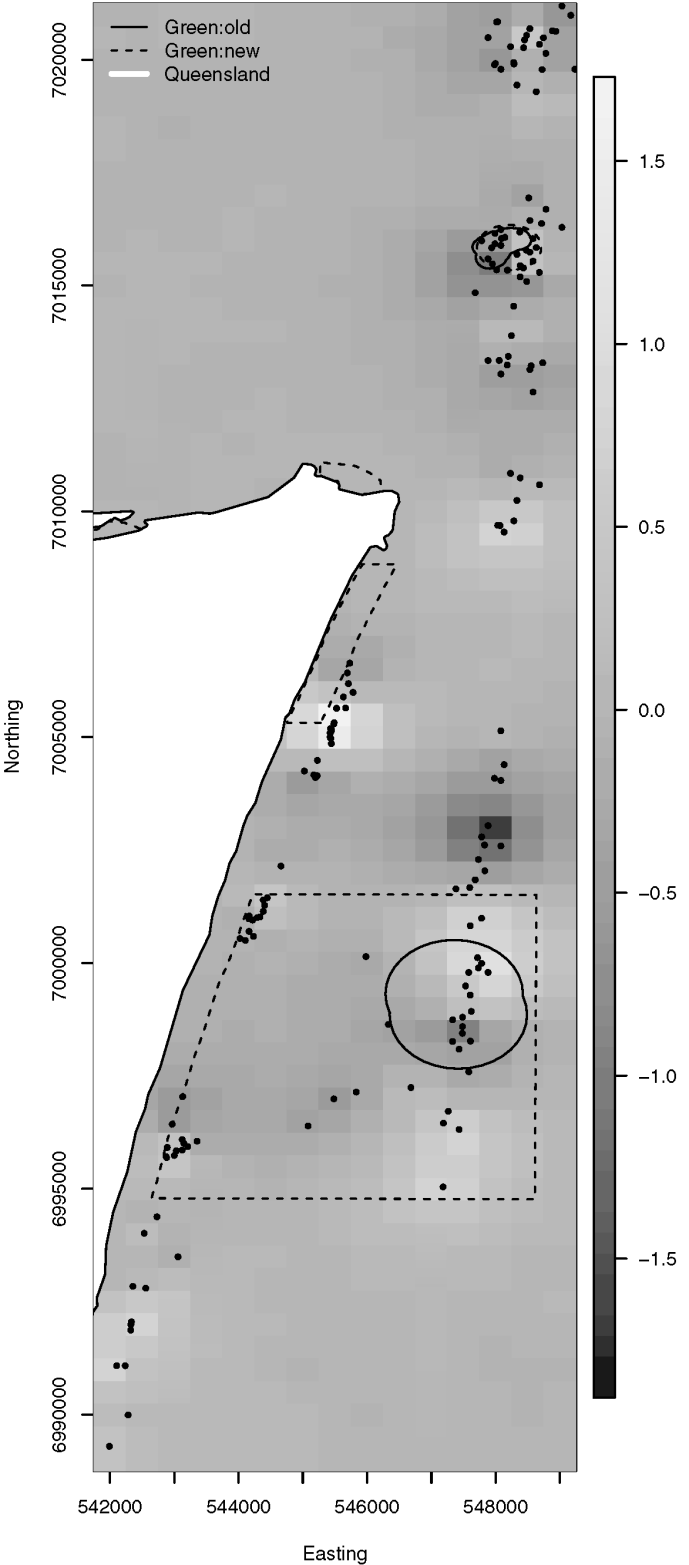
Posterior mean spatial random effects. Each grid cell is 500 × 500m.

**Fig 4:**
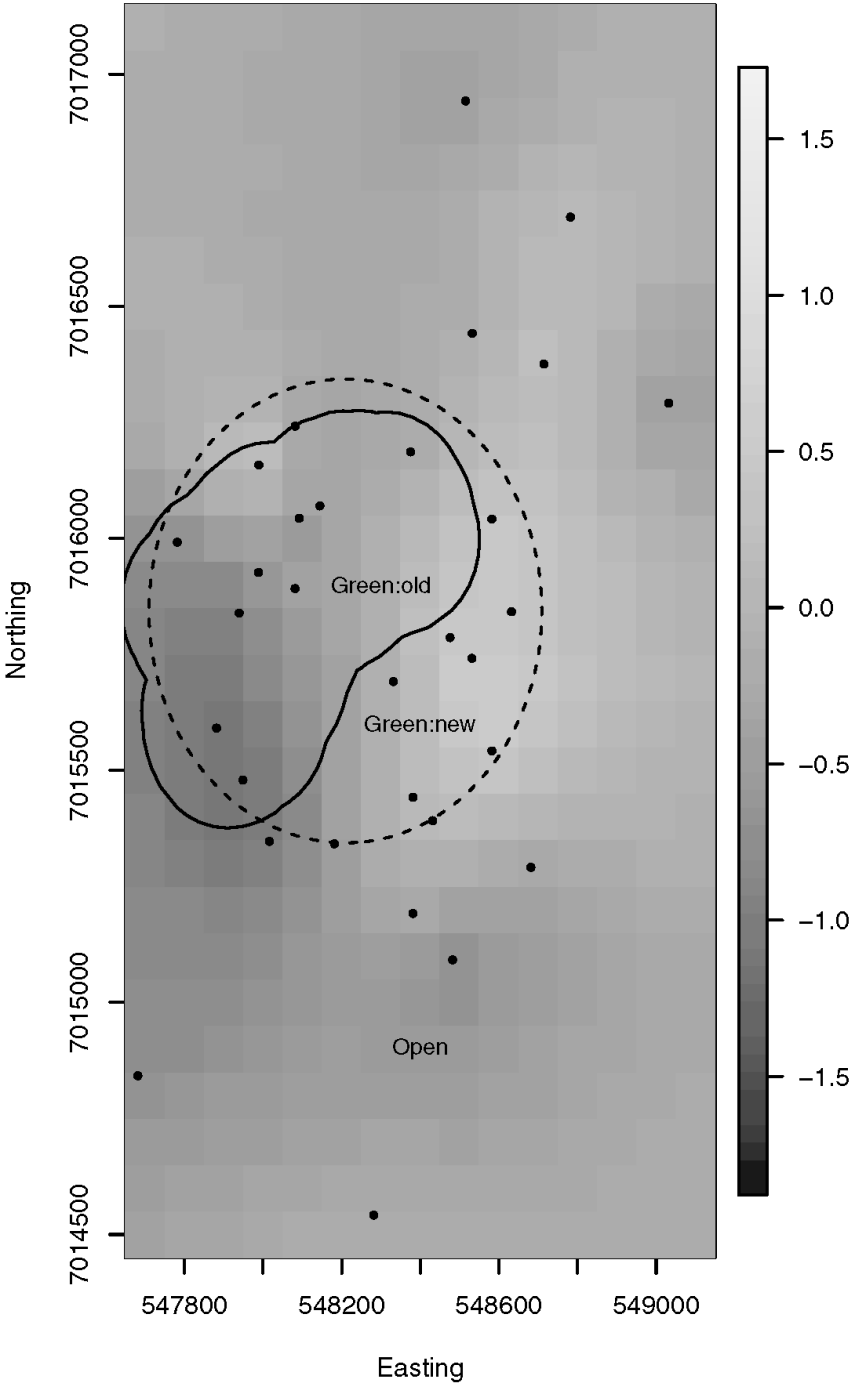
Posterior mean spatial random effects surface for MNP04. Each grid cell is 100 × 100m.

**Fig 5:**
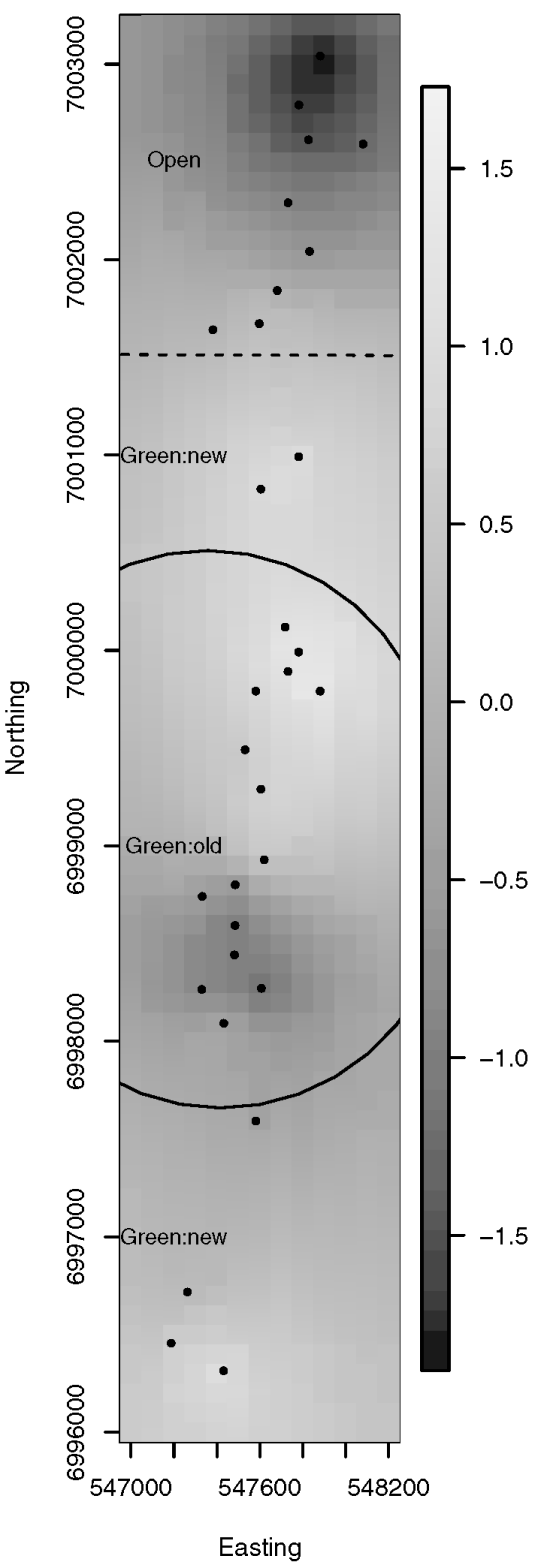
Posterior mean spatial random effects surface for MNP10. Each grid cell is 100 × 100m.

The posterior mean spatial random effects surface is provided in Fig 3. The Queensland coast is provided for context, and the boundaries of new and old green zones are indicated by dashed and solid lines respectively. To provide more detail, Figs 4 and 5 plot the posterior mean spatial effects surfaces from the Spatial Model for subregions of MNP04 and MNP10, respectively. In each of these plots a positive value suggests an adjustment towards a higher probability of snapper presence than described by the covariates, while a negative value suggests an adjustment towards a lower probability of snapper presence. The local structure induced by the spatial correlation can be readily observed by noting the localized regions of positive and negative adjustment. In addition, the spatial random effects appear more variable in MNP10 than in MNP04; i.e., the largest and smallest random effects in MNP10 are more extreme than those for MNP04. This suggests that there may be some unaccounted for features that are more prevalent or more variable in MNP10 than they are in MNP04.

### Predictive capability and model comparison

#### Within sample

We compared the within-sample predictive capabilities of the two models. The posterior mean summary statistics LL, MSE and AUC are provided in Table 2 with accompanying 95% credible intervals. The Spatial Model exhibits a lower MSE as well as a higher LL and AUC than the Non-Spatial Model, although the credible intervals overlap for both MSE and LL. Overall this suggests that the Spatial Model is a better fit for the data within-sample, but not substantially so.

**Table 2:** Posterior mean (95% Credible Intervals) for LL, MSE and AUC under the Spatial and Non-Spatial Models.

Model	LL	MSE	AUC
Non-Spatial Model	-360.39 (-398.46, -340.19)	0.23 (0.21, 0.25)	0.74 (0.72, 0.75)
Spatial Model	-321.79 (-360.76, -294.57)	0.20 (0.18, 0.23)	0.81 (0.78, 0.83)

#### Out-of-sample k-fold Cross Validation

We utilized a 5-fold cross validation to compare the two models, examining the sensitivity of the parameter estimates and comparing out-of-sample predictive capability. In each of five fittings one subset or fold of the data was held out and the Spatial and Non-Spatial Models were fitted to the remaining data. These parameter estimates are shown in S3 Fig for the Non-Spatial and S4 Fig and S5 Fig for the Spatial Model. For both models there is minor variability in estimates across the folds, and as noted previously, at times the uncertainty in the parameter estimates is higher in the Spatial Model than in the Non-Spatial Model.

In each of the five fittings the resulting models were used to predict snapper presences and absences for the hold-out fold. Table 3 provides the resulting LL, MSE and AUC under each of the fittings. Although there is variability across folds, the non-spatial model appears to perform slightly better on the out-of-sample data. That is, the lower posterior mean estimates for LL and MSE, and higher estimates for AUC, are shown for the non-spatial model. These results consistently suggest better cross-validation performance for this simpler model. However, the associated credible intervals (not shown, for brevity) overlap substantially for all three summary statistics. Note, the spatial distribution of the BUV drop sites is quite uneven, particularly for individual folds of the data. As a result the hold-out data are spatially sparse, reducing the ability to take advantage of spatial correlation when predicting at unobserved locations. This characteristic combined with the fairly short estimated spatial range of spatial dependence in the Spatial model may explain the relatively strong performance of the Non-Spatial Model in the cross-validation.

**Table 3:** Posterior mean out-of-sample Log likelihood (LL), Mean Squared Errors (MSE) and Area Under the Curve (AUC) for the k-fold cross validation based on fitted parameter estimates under the Spatial and Non-Spatial Models. Lower estimates of LL and MSE correspond to better predictive performance by a model; higher estimates of AUC corresponds to better predictive performance.

Criterion	Model	Fold 1	Fold 2	Fold 3	Fold 4	Fold 5	Mean
LL	Non-Spatial	-83.12	-65.05	-70.42	-56.28	-99.34	-74.84
	Spatial	-91.00	-73.00	-77.19	-65.92	-106.90	-82.80
MSE	Non-Spatial	0.28	0.21	0.23	0.20	0.26	0.24
	Spatial	0.28	0.23	0.25	0.23	0.27	0.26
AUC	Non-Spatial	0.65	0.77	0.69	0.76	0.69	0.71
	Spatial	0.65	0.71	0.66	0.72	0.68	0.68

## Discussion

The zoning of areas of protection within marine parks requires the consideration of multiple factors including habitat and associated species assemblages [50]. The distribution of sites within the Moreton Bay Marine park relative to habitat presence and green zone boundaries (Fig 1) complicate the assessment of habitat preference. Previous studies have associated snapper with kelp and rocky reefs, but sandy habitat can also be important [21, 23] and strong habitat preferences were not previously observed in Moreton Bay for young age classes [25]. Nevertheless, there is evidence in Moreton Bay that coffee rock habitats may be targeted by some fishers [51]. This latter habitat is extensively distributed along the coast of southeastern Queensland [40] and so it is important to determine whether the BUV data suggest any habitat association with snapper. We used logistic models of snapper presence/absence to assess the degree of habitat preference for sand, kelp, rock substrate and coffee rock and control for other possible sources of variation. In this study, the distribution of snapper, a commercially and recreationally important species of fish, was observed at BUV deployments more often in areas with hard substrate and kelp. In particular, coffee rock, a regionally important substrate formed of Pleistocene sandstone, was the hard substrate most likely for snapper to be observed. The strong relationship between snapper presence and kelp and coffee rock persisted after controlling for the spatial placement of Marine National Park zones (MNPs; i.e., no take areas or “green” zones), environmental factors such as depth, season and visibility, and spatial dependence that might result from other unknown factors. The results therefore suggest that managers seeking to spatially manage resources for the conservation of snapper consider the distribution of these habitats in their marine planning.

The uneven BUV site placement, which was driven by habitat features and management boundaries (Fig 1), requires a model based analysis to control for geographic location. In this particular study, the spatial spread of the BUV deployment sites consists of several subregions where a high density of sites were sampled, with few sites located between the subregions. Furthermore, because of the elongated shape of available submerged reefs, the sites were located in a very linear region along the coast of Moreton Island. Moreover, unobserved and un-modelled covariates may introduce spatial dependence that, if present and unaccounted for, could produce biased and misleading results. Indeed the results suggested strong spatial dependence at short spatial scales (S2 Fig), and incorporating spatial dependence did suggest a few areas with greater, and lesser, probabilities of presence after accounting for the above discussed covariates (note the spatially aggregated dark and light coloured pixels visible in Figs 3, 4, 5). Nevertheless, the predictive capabilities of the two models were similar overall (Tables 2 and 3) and results for habitat associations were not affected (S1 Table and S1 Fig).

Both models with and without spatial dependence revealed a strong positive association between the presence of snapper and the presence of kelp and hard substrate. The Non-Spatial model indicated that the odds of snapper presence increased by almost 10 times where kelp was present, and the Spatial Model estimated a 13 times increase in the odds of presence (based on posterior mean estimates). These are very large effects and are likely far greater than any population recovery expected in the short-medium term attributed to protection zones. A previous study [52] reported a seven fold increase in the number of snapper after four years of protection in New Zealand, although they report on abundance rather than the presence-absence of snapper. Although snapper use sandy habitats this is thought to be associated with lower density populations. Sand was not clearly associated with snapper presence or absence. The models show the odds of snapper presence being around 2 times higher on sites with rock present and around 3 times higher on sites with coffee rock present (S1 Table and Fig 2). The mostly positive but weak relationship with rocky substrate is consistent with previous observations [22]. The strong positive association with the presence of coffee rock and snapper is a new result to the best of our knowledge.

Other predictors in addition to habitat type were found to be important. Depth was found to have a mostly positive influence on snapper presence, although the overall effect was weak. This effect is in line with the major snapper fishing grounds being situated in offshore areas in depths between 20 and 100 metres with larger snapper sometimes being taken in water over 150 metres in depth [22]. Winter likewise had a mostly positive, but weak, positive influence. Commercial and recreational catches of snapper are highest during winter and it is thought that this is because the fishery concentrates on spawning aggregations at this time [22]. Visibility had a positive relationship with snapper presence. This was most likely an observational influence of increased ability to positively detect and identify the species in clear waters.

Old and new green zones were not strongly associated with snapper presence (Fig 2). Although this result is not surprising for the newer green areas, which had only been in place a short amount of time by the end of this study, the older green zones had been in place for more than twelve years. Some reasons for the lack of association include [6]: reserves not big enough [10], lack of enforcement [53–55], protection duration not long enough [56–58] and this analysis only used presence/absence data. The MNP04 green zones, both old and new, were characterised by rocky substrate and an absence of kelp, making them a good but not the best habitat for snapper as indicated by the models. The open areas used as the control for this MNP lack kelp but do contain some coffee rock, associated with an increased presence of snapper. In contrast, all sites in MNP10 (Fig 1) older green zone contained kelp, and the model results indicate that this may be a preferred habitat for snapper. There was also some kelp in the newer green zones, as well as the open areas used as controls. Both coffee rock and rock were found in the green old, green new and open areas. Overall, this suggests a more diverse and preferable habitat for snapper in MNP10 compared to MNP04.

The spatial model provides further information on the spatial dependence through the spatial variance *σ* ^2^ and range parameter *ϕ*. The effective range, which is a function of *ϕ*, is defined as the distance where the spatial correlation drops to negligible levels (see Materials and Methods). For these data, after accounting for other covariate information, the fitted model indicates that the median effective range is approximately 1950m. We do not encourage readers to use this as a specific guideline, however it suggests that previous studies using baited cameras that have used rules of thumb to set minimum distances between sites ranging from 250 to 400m [31, 36] may be an underestimate for species such as snapper. This is not necessarily a problem provided spatial autocorrelation is considered during the survey design and analysis. Some green zones are small and in such instances separating sample sites by large distances may remove the need to account for spatial autocorrelation but would also reduce the potential sample size. It is also conceivable that dependence may differ along north-south distances compared to east-west distances, but this would be difficult to detect within this sampling scheme. Instead, a depth covariate was included in the modeling to offset this concern, since depth is likely to vary differently north-south versus east-west off the eastern coast of Moreton Island. The spatial variance and range parameter often exhibit posterior dependence, which complicates their interpretation, and so these estimates should *not* be interpreted as a minimum distance to be used when picking locations for new studies. Necessary distances to achieve independence at a new site will likely depend on microhabitat factors, and be species specific, that cannot be adequately assumed a priori. Instead, we interpret these results as an indication that spatial correlation exists and should be accounted for when analyzing these kinds of data.

In this study we explored the relevance of marine park boundaries and preferred habitats of *Chrysophrys auratus* in Moreton Bay Marine Park during a period when new no-take areas were introduced. We incorporated the spatial dependence among BUV sites into the modelling framework to test whether the incorporation of highly correlated environmental covariates or the geographic placement of BUV sites produced spatial dependence, but found the spatial term had little effect on the model fit or parameter estimates. Strong positive relationships between the presence of snapper and kelp, coffee rock and rock habitat reinforce the consideration of habitat availability in marine reserve design and the design of any associated monitoring programs. In particular, monitoring programs must consider all major potential habitats, even those that are thought not preferred such as sand for snapper, so as to enable inference on how a species distribution depends on habitat preference across the managed marine landscape. Extending this modelling framework to other species and count data would provide some insight into the general applicability of these results and in particular reveal whether the current practices for the placement of BRUVs are best practice.

## Supporting Information

S1 Table Summary of results for the fitted Non-Spatial Model and Spatial Model. Includes parameter estimates (posterior means) and central 95% credible intervals. ‘*’ indicates significance at the 0.05-level or better.
(PDF)

S1 Fig Estimates for the coefficients in the Spatial Model. Posterior means and accompanying 95% credible intervals.
(EPS)

S2 Fig Prior and posterior for the effective range, 3/*ϕ*, and spatial variance, *σ* ^2^, in meters. (EPS)

S3 Fig Parameter estimates under the five subsets in the Non-Spatial k-fold cross validation. Posterior means and accompanying 95% credible intervals.
(EPS)

S4 Fig Parameter estimates under the five subsets in the Spatial k-fold cross validation. Posterior means and accompanying 95% credible intervals.
(EPS)

S5 Fig Spatial parameter estimates for *σ* ^2^ and *ϕ* under the five subsets in the k-fold cross validation. Posterior means and accompanying 95% HPD credible intervals.
(EPS)

S1 Code Data and R Files. Data is provided as a csv file in S1A. Code for fitting the model is provided using JAGS in S1B. R code for running the model on the provided data is in S1C.
(ZIP)
