# Supplementary material for: Assessing Habitat Use by Snapper (Chrysophrys auratus) from Baited Underwater Video Data in a Coastal Marine Park
Source: PLoS One. 2015 Aug 28;10(8):e0136799. doi: 10.1371/journal.pone.0136799 (PMC4552837; doi:10.1371/journal.pone.0136799)
Supplement: S1 Table — Includes parameter estimates (posterior means) and central 95% credible intervals. ‘*’ indicates significance at the 0.05-level or better. (PDF) [file pone.0136799.s001.pdf]

S1 Table

Summary of results for the fitted Non-Spatial Model and Spatial Model. Includes parameter estimates (posterior means) and central 95% credible intervals. '*' indicates significance at the 0.05-level or better.

	Non-Spatial Model			Spatial Model		
	Est	95%		Est	95%	
(Intercept)	-1.77	(-2.52, -1.05)	*	-2.18	(-3.24, -1.20)	*
Green	-0.45	(-1.03, 0.13)		-0.35	(-1.36, 0.72)	
Green:New	-0.15	(-0.65, 0.35)		-0.12	(-0.81, 0.56)	
Depth	0.27	(0.07, 0.48)	*	0.19	(-0.10, 0.48)	
Rock	0.51	(0.03, 1.01)	*	0.93	(0.22, 1.71)	*
Kelp	2.25	(1.64, 2.87)	*	2.59	(1.52, 3.75)	*
Sand	-0.04	(-0.52, 0.44)		-0.27	(-0.99, 0.41)	
Coff	1.13	(0.65, 1.60)	*	1.28	(0.56, 2.01)	*
Winter	0.62	(0.25, 1.00)	*	0.72	(0.31, 1.13)	*
Visibility	0.30	(0.09, 0.52)	*	0.31	(0.07, 0.55)	*
ϕ				0.002	(0.001, 0.005)	
σ^2				1.33	(0.59, 2.70)	
